# Epidemiological Study of Return to Work and Mortality in Lung Cancer Survivors

**DOI:** 10.3390/ijerph19010309

**Published:** 2021-12-28

**Authors:** Zhe-Yu Yang, Ching-Huang Lai, Ching-Liang Ho, Chung-Ching Wang

**Affiliations:** 1 Division of Family Medicine, Department of Family and Community Medicine, Tri-Service General Hospital, National Defense Medical Center, Taipei 114, Taiwan; yang820731@gmail.com; 2 School of Public Health, National Defense Medical Center, Taipei 114, Taiwan; lgh@mail.ndmctsgh.edu.tw; 3 Division of Hematology/Oncology, Department of Medicine, Tri-Service General Hospital, School of Medicine, National Defense Medical Center, Taipei 114, Taiwan; charileho22623@gmail.com; 4 Division of Occupational Medicine, Department of Family & Community Medicine, Tri-Service General Hospital, National Defense Medical Center, Taipei 114, Taiwan

**Keywords:** lung cancer, return to work, survival outcome

## Abstract

Lung cancer is the second most common cancer and the leading cause of cancer-related deaths worldwide. Return to work (RTW) plays an important role for lung cancer survivors. Few studies focus solely on the relationship among possible variables and the RTW of lung cancer patients. The aim of our study was to examine sociodemographic, disease-related and work-related factors associated with RTW among lung cancer survivors in Taiwan. A total of 2206 employees who had been diagnosed with lung cancer at the Labor Insurance Database (LID), Taiwan Cancer Registry (TCR) and the National Health Insurance Research Database (NHIRD) during the period 2004–2015, were included in the study. We used the Cox proportional hazards model to investigate the associations between sociodemographic, disease-related and work-related factors on one hand and RTW on the other hand. The Kaplan–Meier method was used for analyzing the survival probability. Patients with an early cancer stage and those who underwent surgery had a higher likelihood of RTW. Factors including older age, male, higher monthly income and receipt of radiotherapy were inversely correlated with RTW. For lung cancer patients, RTW was a predictor of a lower risk of all-cause mortality in both the unadjusted and fully adjusted model. A better survival rate was found in stage III and IV lung cancer patients who had RTW. Sociodemographic and clinical-related variables had an impact on RTW among employees with lung cancer. RTW was correlated with a lower risk of all-cause mortality and better lung cancer survival. Our study showed the influence of RTW and independent confounding factors in lung cancer survivorship.

## 1. Introduction

Lung cancer is the second most common cancer and the leading cause of cancer-related deaths in both sexes worldwide [1]. A total of 235,760 estimated new cases of lung cancer and 131,880 deaths from lung cancer were reported in the US in 2021 [2]. Lung cancer accounted for 19% of cancer deaths in Taiwan in 2017. Lung cancer mortality rates are high and overall 5-year survival rates are 21% [2]. Tobacco smoking, occupational exposures, air pollution and genetic predisposition are risk factors of lung cancer [3]. In Asia, exposure to indoor air pollution from cooking plays a crucial role in female lung cancer [4]. Traditional treatments for lung cancer included surgery, chemotherapy and radiotherapy [5]. In the past two decades, targeted therapies have been developed and approved as first-line therapies in lung cancer [6]. Immunotherapy had also been developed as a possible treatment option [7].

Mortality rates from lung cancer have been dropping due to lung cancer screening and novel treatment strategies [8,9]. However, lung cancer survivors still face many issues, including physical disability, psychological distress and social relationship problems [10,11]. Patients experienced pain, fatigue, weakness, shortness of breath and exercise intolerance [12]. Depression and anxiety were also present in patients with lung cancer [13,14]. The cost of cancer treatment and the loss of employment after a lung cancer diagnosis might cause economic problems [15]. Emerging studies have discussed the high financial burden that cancer patients face [16,17]. Moreover, a prior study reported that the unemployment rates of patients with lung cancer were two-fold higher than that of the general population [18]. Thus, the impact of lung cancer on RTW is worthy of attention.

Previous studies have investigated the correlation between the RTW of lung cancer survivors and variable factors [19,20]. However, prior research included limited samples of lung cancer survivors (<500 subjects). A paucity of studies investigated the relationship between RTW and survival rate for lung cancer patients. The objective of our retrospective cohort study was first, to explore the demographic, clinical and occupational variables correlated with RTW and second, to examine the association between RTW and survival rate among lung cancer survivors in Taiwan.

## 2. Materials and Methods

### 2.1. Study Population and Database

**Figure 1:**
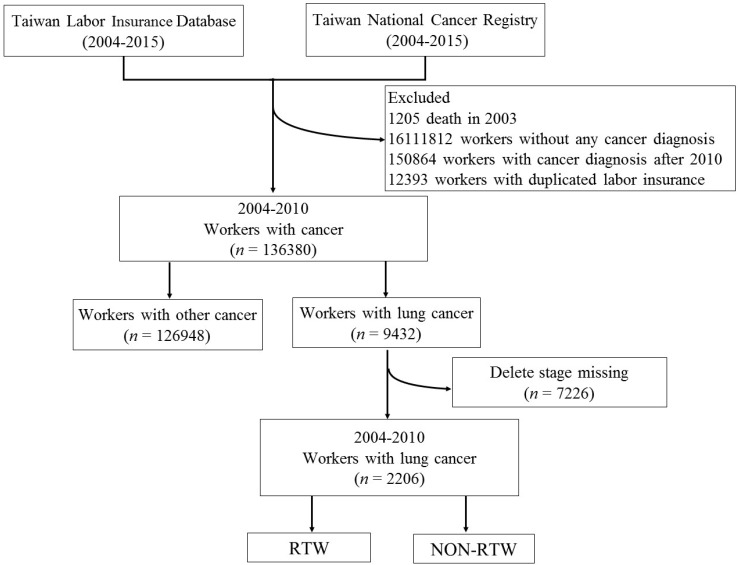
Flow chart of study population.

During the period 2004–2015, 2206 employees were newly diagnosed with lung cancer (ICD-O-3); information on these employees was collected from the Taiwan National Cancer Registry (TNCR) and the Taiwan Labor Insurance Database (LID) for this retrospective cohort study. Detailed descriptions are summarized in Figure 1. According to the International Classification of Diseases for Oncology—3rd edition (ICD-O-3), a primary diagnosis of lung cancer is coded. We utilized the unique encryption identity number to link these 2 databases in order to obtain sociodemographic information, such as age, employment information, employee’s industry, monthly income and company size. Disease-related information, including primary cancer site, cancer stage and types of treatment, were also collected. The exclusion criteria of our study were (1) death before enrolled period; (2) patients who had lung cancer combined with other cancer or unknown timing of lung cancer diagnosis; (3) lung cancer patient who was unemployed at baseline; (4) lung cancer diagnosis after 2010. The study protocol was reviewed by the Institutional Review Board (IRB) of Tri-Service General Hospital (TSGH) (IRB No. 1-107-05-129).

### 2.2. Sociodemographic and Disease-Related Information

Sociodemographic data from the LID included age, monthly income, employment data, employee’s industry and company size. In accordance with the International Classification of Diseases, Ninth Revision, Clinical Modification (ICD-9-CM) codes, clinical comorbidities were identified from National Health Insurance Research Database (NHIRD), including disorders of lipid metabolism, alcohol abuse, cerebrovascular diseases, chronic pulmonary diseases, peptic ulcer diseases, renal diseases, liver diseases and depression. Supplemental Table S1 lists the ICD-9-CM codes for clinical comorbidities. We also collected disease-related data, such as types of treatment and the pathological stage of lung cancer from TNCR and NHIRD.

### 2.3. Outcome Assessment

RTW was defined as employees who have still-insured labor insurance or first time re-insured labor insurance within 1 year of their lung cancer diagnosis. Non-RTW was defined as employees who exited labor insurance within 1 year of their lung cancer diagnosis and did not re-insure again. We selected an RTW period of 1–5 years after their first lung cancer diagnosis. We identified the information regarding RTW from employment data within LID. Every eligible participant was traced from their first-time primary diagnosis of lung cancer to the date of their death or the end of their follow-ups. We set the all-cause mortality (death from all causes) within the period 2004–2015 as the study endpoint.

### 2.4. Statistical Analysis

PROC PHREG of the SAS statistical package (version 9.4, SAS Institute Inc., Cary, NC, USA) was utilized to perform our data analyses. Two-sided *p* values < 0.05 were considered to be statistically significant. RTW was determined from the data of their first primary diagnosis of lung cancer to the reemployment date within five years. Survival time was calculated from the data of their first primary diagnosis of lung cancer to the date of their death during the follow-up period (2004–2015). The results of statistical analyses are described as the means, standard deviations (SD) and percentages. Differences of categorical variables between subgroups were analyzed by using a chi-squared test. Continuous variables were compared by using an independent *t*-test.

Cox regression is a technique for evaluating the association between variables and specific events [21]. We used a multivariable Cox proportional hazard regression as a statistical model to assess (1) the impact of confounding factors on RTW and (2) the relationship between all-cause mortality and RTW among lung cancer patients. Covariates including age, gender, pathological stage of lung cancer, received treatment, monthly income, employee’s industry and company size were adjusted. The HR (hazard ratio) represented the chance of RTW. Lastly, we used the Kaplan–Meier method to analyze the survival probability [22] and log-rank test to differentiate the survival curves between RTW and non-RTW groups.

## 3. Results

### 3.1. Characteristics of the Study Population

The sociodemographic characteristics of the study population are shown in Table 1. The mean age of the participants was 53.5 ± 8.2 years. Of the 2206 patients, 1095 (49.6%) workers were male and 1109 (50.2%) workers had a monthly income range below USD 960. In this study, the early stage (stage 0 and I) of lung cancer were in the majority (48.4%), followed by stage IV (20.3%), stage III (19.6%) and stage II (11.6%). A total of 1805 (81.8%) employees received surgical intervention, while less than half of the workers received chemotherapy (39.4%) or radiotherapy (15.5%). The employment rates of lung cancer survivors 2 and 5 years after their RTW were 60.3% and 41.1%, respectively.

### 3.2. Univariate Analysis of Independent Factors Associated with RTW in Cox Proportional Hazards Models

**Figure 2:**
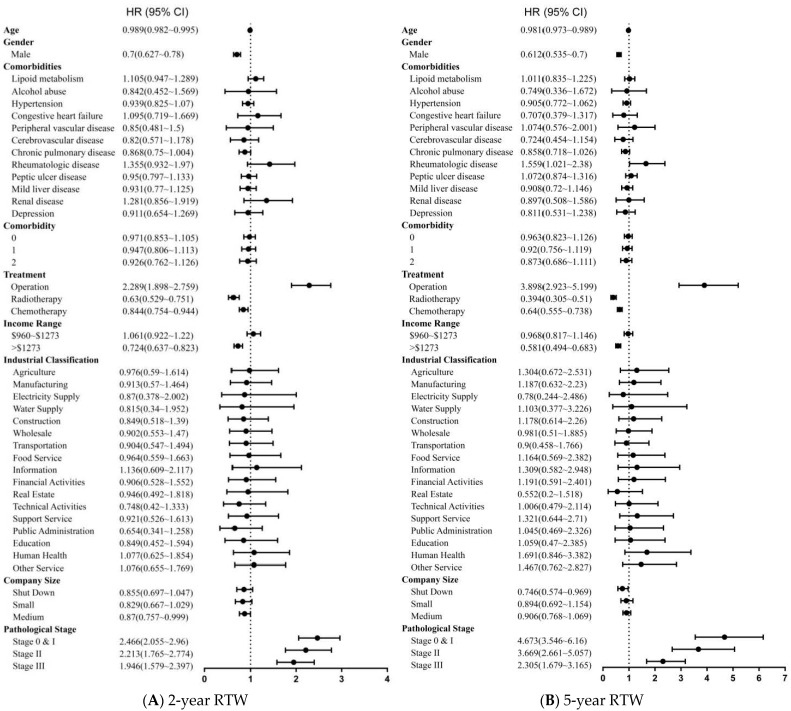
Forest tree plot showing the HR obtained by univariate Cox proportional hazards model of return to work for lung cancer survivors in (**A**) 2-year RTW and (**B**) 5-year RTW.

Figure 2 shows the independent factors associated with RTW using univariate Cox proportional hazards models. A lower likelihood of RTW was associated with older age, male and a monthly income above USD 1273. The patients who received chemotherapy or radiotherapy were less likely to RTW. These two HRs gradually declined during the follow-up period (chemotherapy: second year HR = 0.84; 95% CI = 0.75–0.94 to fifth year HR = 0.64; 95% CI = 0.55–0.73; radiotherapy: second year HR = 0.63; 95% CI = 0.52–0.75 to fifth year HR = 0.39; 95% CI = 0.3–0.51). A greater likelihood of RTW was associated with early stage cancer and receiving surgery.

### 3.3. Multivariate Analysis of Independent Factors Associated with RTW in Cox Proportional Hazards Models

**Figure 3:**
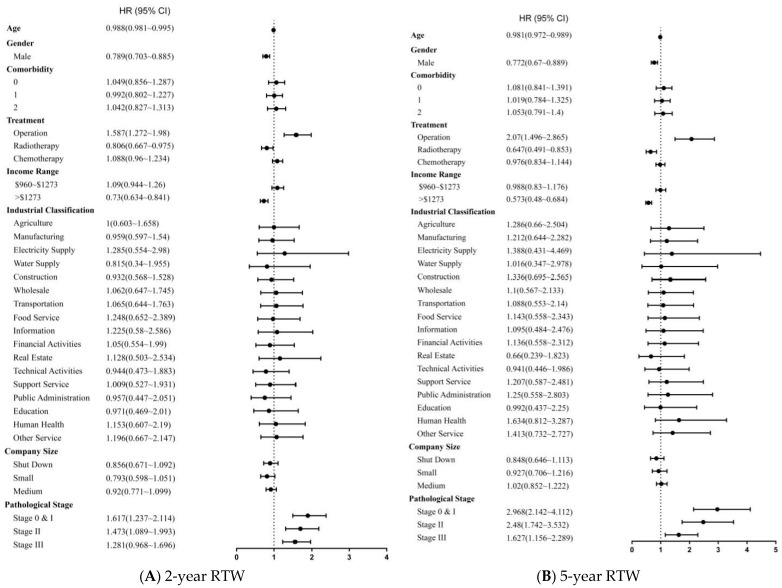
Forest tree plot showing the HR obtained by multivariate Cox proportional hazards model of return to work for lung cancer survivors in (**A**) 2-year RTW and (**B**) 5-year RTW.

An examination of the correlation of RTW and independent factors using multivariate Cox proportional hazards models suggested that the factors correlated with a lower likelihood of RTW were older age, male, a monthly income above USD 1273 and receiving radiotherapy (Figure 3). The patients who received surgery had a higher chance of RTW during the study period. Compared with the participants with stage IV, the participants with stage 0, I, II or III had a higher chance of RTW.

### 3.4. Survival Rates by RTW and Non-RTW

**Figure 4:**
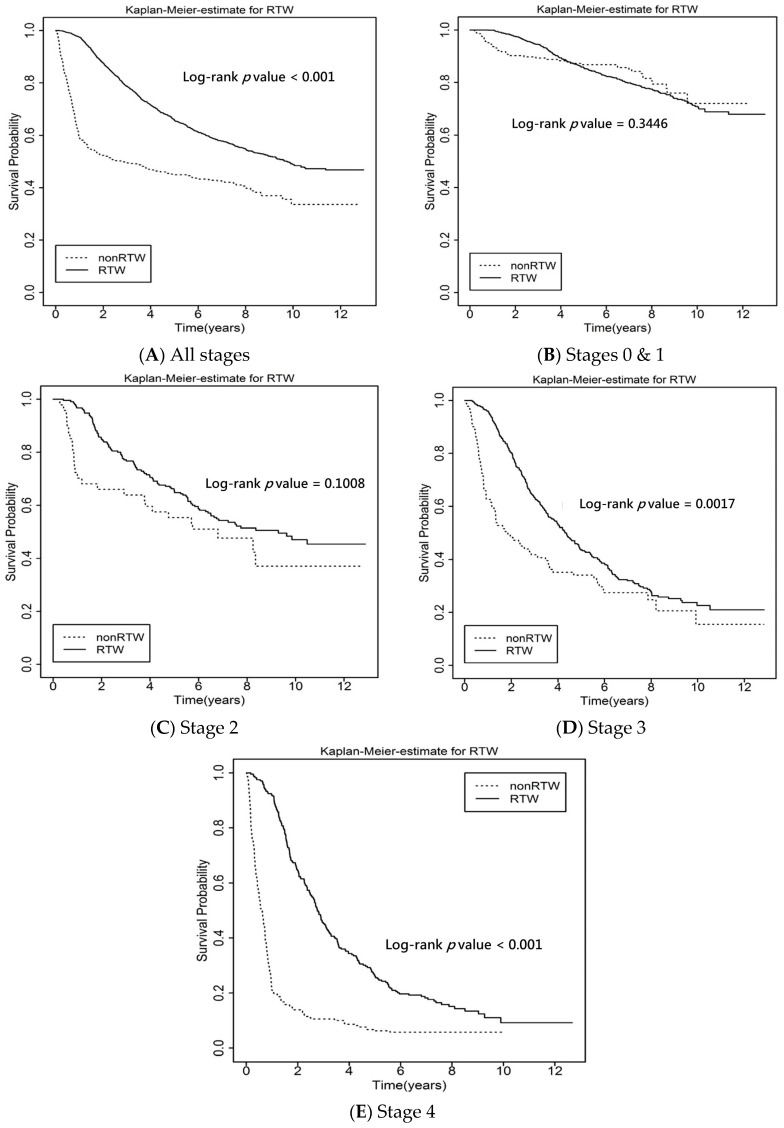
Kaplan–Meier (KM) curves showed survival probability of RTW for (**A**) all, (**B**) stages 0 and 1 and (**C**–**E**) stages 2–4 lung cancer survivors.

The survival rates using the Kaplan–Meier curve stratified by RTW and non-RTW are listed in Figure 4. The patients who had RTW had a better survival probability than those who had not RTW in all the cases of lung cancer (*p* < 0.001) and especially for patients with stage III and IV (*p* < 0.01).

### 3.5. Multivariate Analysis of RTW and All-Cause Mortality in Cox Proportional Hazards Models

After using Cox proportional hazards models to adjust the confounding variables, the association of all-cause mortality between lung cancer survivors who had RTW and those who had not is shown in Table 2. An inverse relationship between all-cause mortality and RTW among lung cancer patients was observed in both the unadjusted and fully adjusted model (*p* < 0.001; *p* < 0.001).

## 4. Discussion

Our study investigated the independent factors associated with RTW and examined the correlation between all-cause mortality and RTW among lung cancer patients in Taiwan. We found that employees with stages 0, I and II cancer had a higher likelihood of RTW, while factors correlated with a reduced likelihood of RTW were higher income and receiving radiotherapy. Lung cancer survivors who had RTW had lower all-cause mortality rates than those who had not. Moreover, through the Kaplan–Meier curve, the RTW group had better survival rates than the non-RTW group.

There have been many studies examining the association between RTW and cancer survivorship [23]. In a systemic review, Mehnert reported that 40% of patients had returned to work or had continued to work after 6 months of treatment, 62% by 12 months and 89% by 24 months [24]. However, the RTW rate of cancer patients was widely different depending on cancer type. A prospective study demonstrated that patients with breast cancer or skin cancer had a higher RTW rate. On the contrary, patients with lung cancer or head and neck cancer had a lower RTW rate [25]. Another study showed patients with skin or stomach cancer had higher employment rates compared to patients with lung or central nervous system cancer [26]. Earle and his colleagues reported only 21% of lung cancer survivors remained employed in the 15 months after their cancer diagnosis [27]. A recent cross-sectional study from Germany revealed a 33% employment rate within 1 year after being diagnosed with lung cancer [28]. Our study found a 41% employment rate in the fifth year of the follow-up period. The possible reasons for the relatively low RTW rate of lung cancer survivors have been addressed. A review article on the employment-related factors of cancer survivors suggested that the poor 5-year survival rates of lung cancer patients might cause the low likelihood of RTW [29]. Polanski et al. stated that lung cancer survivors had a lower quality of life compared to other cancer survivors [10].

Sociodemographic factors including age, education and income were examined to be associated with RTW [26]. A prospective study in the US indicated that a lower educational level and income were associated with unemployment [27]. Kim and his colleagues found that a low employment rate was correlated with older age and lower household income among lung cancer survivors [18]. However, our study revealed that a higher monthly income was correlated with a lower likelihood of RTW, which is inconsistent with previous studies. There are several explanations for these conflicting findings. The Taiwanese National Health Insurance system has provided comprehensive coverage, including the medical expense of cancer treatment [30]. The coverage rate of the Taiwanese National Health Insurance is nearly 99.9%, and around 93% of medical care institutions in Taiwan are contracted with the National Health Insurance system. Taken together, cancer patients in Taiwan face less financial stress on treatment costs. From an economic perspective, the higher monthly income group might have better financial reserves that allow them to take long-term sick leave.

In the present study, we found that lung cancer survivors who had RTW had higher survival rates and lower risk of all-cause mortality. An American review stated that RTW could help to improve recovery in many aspects, such as social, psychological and physical functioning [31]. Schmidt et al. discovered that participants who RTW have a better quality of life and cognitive performance than those who did not RTW [32]. A cross-sectional study from a single cancer center demonstrated that cancer survivors who remained in their jobs had fewer mental illnesses compared with those were out of work [33]. According to these findings, re-employment in cancer patients might indicate the recovery of physical function and improvement of cancer survival. Our results are in line with this inference.

There are several limitations in our study. First, there was a lack of information about other sociodemographic factors and psychosocial work-related factors, which may be possible confounding factors affecting the RTW. Second, we only recruited Taiwanese participants, which might limit the generalizability of our findings to different racial populations. Third, we did not take into account the side effects of treatment, such as fatigue, which was the most common work-related problem in lung cancer patients.

## 5. Conclusions

Our study showed RTW was correlated with a lower risk of all-cause mortality and better lung cancer survival. The findings might be explained by a stable income and better performance status accompanied by RTW. Our results demonstrated the impact of RTW and independent confounding factors in lung cancer survivorship. Accessing the side effects of treatment, quality of life, work accommodation and work discrimination may be considered in future studies.

## Figures and Tables

**Table 1:** Characteristics of the study population.

Characteristic	Total		RTW Group			Non-RTW Group		
	Number of Patient	%	2-Year RTW *n* = 1332	5-Year RTW *n* = 908	*p* Value	2-Year RTW *n* = 874	5-Year RTW *n* = 1298	*p* Value
Age(year) ^a^	53.5 ± 8.2 (26–91)	-	52.8 ± 8 (26–81)	52.3 ± 8 (26–81)		54.6 ± 8.4 (26–91)	54.4 ± 8.2 (26–91)	
Gender								
Male	1095	49.63	553	346	0.1059	542	749	0.0449
Comorbidities								
Lipoid metabolism	289	13.1	188	120	0.5445	101	169	0.3106
Alcohol abuse	20	0.9	10	6	0.804	10	14	0.886
Hypertension	501	22.71	289	191	0.708	212	310	0.8417
Congestive heart failure	34	1.54	22	10	0.2812	12	24	0.3942
Peripheral vascular disease	23	1.04	12	10	0.6368	11	13	0.5741
Cerebrovascular disease	60	2.71	30	18	0.665	30	42	0.8017
Chronic pulmonary disease	396	17.95	215	144	0.8582	181	252	0.4589
Rheumatologic disease	36	1.63	28	22	0.6138	8	14	0.7094
Peptic ulcer disease	240	10.87	139	104	0.4467	101	136	0.4292
Mild liver disease	206	9.33	117	78	0.8733	89	128	0.8063
Renal disease	32	1.45	24	12	0.3749	8	20	0.2051
Depression	65	2.94	36	22	0.6823	29	43	0.9946
Comorbidity								
0	1097	49.72	676					
1	578	26.2	348					
2	322	14.59	189					
≥3	209	9.47	119					
Treatment								
OP	1805	81.82	1211	859	0.0012	594	946	0.0133
RTB	343	15.54	141	62	0.0024	202	281	0.4212
CH	870	39.43	477	272	0.0039	393	598	0.6121
Monthly income (USD)					0.1728			0.312
≤$960	1109	50.27	715	519		394	590	
>$960–$1273	395	17.9	270	182		125	213	
>$1273	702	31.82	347	207		355	495	
Employee’s industry					0.9589			0.9977
Agriculture	154	6.98	97	69		57	85	
Manufacturing	692	31.36	415	290		277	402	
Electricity and Gas Supply	14	0.63	8	4		6	10	
Water Supply	13	0.58	7	5		6	8	
Construction	232	10.51	129	95		103	137	
Wholesale and Retail Trade	254	11.51	152	90		102	164	
Transportation and Storage	168	7.61	99	54		69	114	
Food Service	74	3.35	46	30		28	44	
Information	30	1.35	22	14		8	16	
Financial	82	3.71	50	36		32	46	
Real Estate Activities	29	1.31	18	6		11	23	
Technical Activities	63	2.85	32	23		31	40	
Support Service Activities	63	2.85	38	29		25	34	
Public Administration	40	1.81	18	15		22	25	
Education	36	1.63	21	14		15	22	
Human Health	67	3.03	47	40		20	27	
Arts	28	1.26	18	10		10	18	
Other Service Activities	167	7.57	115	84		52	83	
Company size ^b^					0.8169			0.6518
Company closed	187	8.47	104	62		83	125	
Small	167	7.57	90	65		77	102	
Medium			260	183		200	277	
Large			878	598		514	794	
Pathological stage					<0.0001			0.0586
0 & I	1068	48.41	776	611		292	457	
II	257	11.65	168	116		89	141	
III	433	19.62	252	126		181	307	
IV	448	20.3	136	55		312	393	

Abbreviation: RTW = return to work, OP = operation, RTB = radiotherapy, CH = chemotherapy. ^a^ values are mean (standard deviation). ^b^ Company size: small (less than 5 people), medium (less than 200 people in manufacturing, construction, mining and quarrying; or less than 100 people in other industries), large (more than 200 people in manufacturing, construction, mining and quarrying; or more than 100 people in other industries).

**Table 2:** Uni- and multivariate analysis of RTW and all-cause mortality in Cox proportional hazards models.

	Unadjusted All-Cause Mortality	*p*	Adjusted All-Cause Mortality	*p*
RTW	0.508 (0.447–0.578)	<0.0001	0.508 (0.441–0.586)	<0.0001

## Data Availability

The data underlying this study are from the Labor Insurance Database (LID) and National Health Insurance Research Database (NHIRD). The LID and NHIRD is not free to public access, and therefore, interested researchers can obtain the data through formal application to the Health and Welfare Data Science Center, Ministry of Health and Welfare, Taiwan. (https://dep.mohw.gov.tw/DOS/np-2497-113.html). Last accessed date: 29 June 2020.

## Supplementary Materials

The following are available online at www.mdpi.com/article/10.3390/ijerph19010309/s1, Table S1: The ICD-9-CM codes for clinical comorbidities.
